# Study on Enzyme Activity and Metabolomics during Culture of Liquid Spawn of *Floccularia luteovirens*

**DOI:** 10.3390/jof10090618

**Published:** 2024-08-29

**Authors:** Yanqing Ni, Qiuhong Liao, Siyuan Gou, Tongjia Shi, Wensheng Li, Rencai Feng, Zhiqiang Zhao, Xu Zhao

**Affiliations:** 1 Institute of Urban Agriculture, Chinese Academy of Agricultural Sciences, Chengdu 610299, China; niyanqing0214@163.com (Y.N.); qiu5711@163.com (Q.L.);; 2 College of Food and Biological Engineering, Chengdu University, Chengdu 610106, China; 3 Chengdu National Agricultural Science and Technology Center, Chengdu 610299, China; 4 Zhuoni County Agricultural Technology Extension Station, Gannan 747600, China

**Keywords:** *Floccularia luteovirens*, liquid spawn, enzyme activity, metabolomics

## Abstract

To comprehensively investigate the physiological characteristics and metabolic processes of the mycelium of *Floccularia luteovirens* (*F. luteovirens*), a wild edible fungus unique to the plateau region, we conducted an in-depth analysis of the mycelium enzyme activity and metabolites during different culture periods. The activity of seven enzymes all followed a trend of initially increasing and then decreasing. The intra- and extracellular activity peaks of three hydrolases—amylase, protease, and cellulase—all occurred on the 20th day, except for the extracellular amylase, which peaked on the 15th day. In contrast, the peak activity of laccase occurred on the 10th day. Moreover, three types of oxidoreductases in the mycelium (catalase (CAT), superoxide dismutase (SOD), and 2,3,5-triphenyltetrazolium chloride (TTC)-dehydrogenase (TTC-DH)) also exhibited significant changes in activity. CAT and SOD activity reached their maximum on the 20th day, whereas TTC-DH showed high activity on both the 10th and 20th days. Through a comprehensive assessment of the evolving trends of these physiological parameters, we determined that the optimal cultivation cycle for *F. luteovirens* liquid spawn is 20 days. An untargeted metabolomic analysis revealed that 3569 metabolites were detected in the *F. luteovirens* mycelium, including a variety of secondary metabolites and functional components, with terpenoids being particularly abundant, accounting for 148 types. By comparing three different culture stages (10 days, 20 days, and 30 days), 299, 291, and 381 metabolites, respectively, showed different accumulation patterns in the comparison groups of 10d vs. 20d, 20d vs. 30d, and 10d vs. 30d. These differential metabolites were primarily concentrated in carboxylic acids and their derivatives, fatty acyl groups, organic oxygen compounds, and lipid compounds. In addition, there were several amino acids whose abundance continued to grow during culturing. The metabolism of amino acids greatly affects mycelium growth and development. This research delineates the interplay between mycelium growth and metabolism, offering empirical support for a cultivation strategy for liquid *F. luteovirens*, and an exploration of its metabolites for potential applications.

## 1. Introduction

*Floccularia luteovirens* (*F. luteovirens*), also known as the ghee mushroom or grassland yellow mushroom, is an edible wild mushroom unique to the Qinghai–Tibet Plateau region [1]. Its fruiting body is notable for its high protein content and complete set of 18 amino acids. Furthermore, it contains functional components, such as polysaccharides, selenium, vitamins, flavonoids, glycosides, and sterols [2,3]. This mushroom, used in traditional Tibetan medicine, exhibits anti-inflammatory, antitumor, and immunity-boosting properties [4], making it a promising edible fungus for further research and development. However, artificial cultivation of *F. luteovirens* has not yet been realized, and its mycelial growth rate is extremely slow under artificial conditions. In recent years, many edible mushroom species have been found to exhibit faster growth rates under submerged cultivation conditions and to accumulate a higher biomass and more bioactive metabolites [5]. For instance, common edible fungi, such as *Lentinula edodes*, *Pleurotus ostreatus*, and *Flammulina velutipes*, have established standardized protocols for liquid spawn production [6,7,8]. For species with slow growth rates, such as *F. luteovirens*, the adoption of liquid culture techniques with which to swiftly acquire a substantial amount of mycelia is a crucial prerequisite for artificial cultivation research and applications.

However, control of the liquid culture duration is particularly critical. An insufficient culture time may result in inadequate mycelial biomass with which to meet subsequent cultivation requirements, whereas an excessively prolonged culture period could lead to an excessive accumulation of metabolites, thereby affecting the vitality of the spawn [6,9]. During liquid fermentation, hydrolytic enzymes play an indispensable role by breaking down complex organic matter in the culture medium, thereby providing essential nutrients for mycelial growth [10]. Concurrently, the intracellular antioxidant enzyme system, such as superoxide dismutase (SOD) and catalase (CAT), maintains redox balance, ensures the normal physiological functioning of the cells, and indirectly reflects the health status and degree of aging of the cells [11]. Furthermore, 2,3,5-triphenyltetrazolium chloride (TTC)-dehydrogenase (TTC-DH) activity, as an indicator of microbial metabolic capabilities regarding organic substances, has served as a vital parameter for assessing the vitality of spawn in edible fungi, such as *Auricularia auricula-judae* and *Morchella sextelata* [12,13]. Regrettably, research on changes in enzyme activity during the preparation of liquid spawn and the cultivation process of *F. luteovirens* is currently lacking. This gap in the literature urgently needs to be addressed to comprehensively understand and optimize liquid culture techniques for *F. luteovirens*.

The cultivation of liquid mycelial cultures serves as a vital material not only for artificial cultivation experiments, but also as a conduit for procuring target metabolites from the mycelium. The mycelium itself is a rich source of nutritional value, bearing compositional similarities to the fruiting body [14], and can serve as an alternative substrate, providing proteins, lipids, fatty acids, vitamins, etc. Liquid cultivation techniques are adept at efficiently generating high-quality mycelia, and optimizing the culture medium can significantly enhance production yields [9]. Most importantly, the mycelium derived from fermentation cultivation can be a source of natural products with specific physiological activities, such as extracellular polysaccharides with immunomodulatory effects [15,16], and triterpenes and alkaloids with antitumor and antioxidant properties [17,18]. These research outcomes have revealed the enormous potential of mycelia as a prospective substitute for meat and as a source of therapeutic natural compounds [19,20]. The current body of work on *F. luteovirens* is predominantly confined to comparative analyses of the constituents found within wild fruiting bodies. Conversely, the metabolites derived from the mycelial phase remain largely uncharacterized. The metabolic products within the mycelium are crucial for the synthesis and accumulation of bioactive substances. Metabolomics, a comprehensive analytical approach, is capable of profiling low-molecular-weight metabolites within organisms, directly reflecting the metabolic changes under various conditions [21]. In the study of edible fungi, the application of metabolomics allows for a systematic analysis of the metabolites produced by macrofungi, aiding in the elucidation of their metabolic patterns [22], the screening of bioactive substances [23], and the optimization of cultivation conditions [24]. In this study, the mycelia of *F. luteovirens* cultivated through liquid fermentation at 10, 20, and 30 days were analyzed by employing a non-targeted metabolomic analysis technique combining GC-MS and LC-MS, and a comprehensive examination of the types and quantities of metabolites within the mycelium was conducted. The aim of this study was to explore the patterns of bioactive substance changes within the mycelium, identify the key materials and their metabolic pathways during mycelial growth, and provide a scientific basis for the further cultivation and application of *F. luteovirens*.

## 2. Materials and Methods

### 2.1. Tested Strains

The tested strains were deposited at the Institute of Urban Agriculture, Chinese Academy of Agricultural Sciences, and identified as *F. luteovirens* by ITS.

### 2.2. Main Instruments and Equipment

A ZHTY-50E oscillation incubator (Shanghai Zhicu Instrument Co., Ltd., Shanghai, China), SCIENTZ-50 f/A vacuum freeze-drying apparatus (Ningbo xingzhi freeze-drying equipment Co., Ltd., Ningbo, China), Spark microplate reader (Tecan China (Shanghai) Laboratory Equipment Co., Ltd., Shanghai, China), Eppendorf 5424R microcentrifuge (Ebende (Shanghai) International Trade Co., Ltd., Shanghai, China), Waters ACQUITY UPLC I—Class plus/Thermo QE HF series ultra-high-performance liquid high-resolution mass spectrometer (Thermo Fisher Scientiffc, Shanghai, China), and Agilent 7890 B-5977-B gas chromatography–mass spectrometer (Agilent Technologies, Inc., Beijing, China) were used.

### 2.3. Culture Medium Preparation and Liquid Spawn Culture

The liquid medium formula is as follows: potato dextrose broth culture medium dry powder (Guangdong Huankai Microbial SCI&TECH Co., Ltd., Guangzhou, China), 24 g; NH_4_NO_3_ 3 g and MgSO_4_, 0.3 g; and distilled water, 1000 mL. Each conical flask (with a volume of 250 mL) was filled with 140 mL of the culture medium and sterilized at 121 °C for 20 min until use. For its liquid spawn cultivation, *F. luteovirens* seeding material was broken down into a suspension using a sterilized wall breaker and thoroughly mixed. A pipetting gun was used to accurately add 10 mL of mushroom seeding material into a conical flask with liquid medium. Set the incubator shaker to 25 °C and 125 rpm, then place the shaker inside and cultivate in the dark.

### 2.4. Sample Collection and Physiological Index Measurement

Mycelia were collected every 5 days until the 30th day of culture. After washing the mycelia with distilled water, the mixture was centrifuged at 8000 r/min for 5 min. The supernatant was discarded and the process was repeated three times. The mycelium was then flash-frozen in liquid nitrogen and stored at −81 °C in a freezer for subsequent experiments. The biomass was determined by weighing the mycelium after freeze-drying, the pH was measured directly from the fermentation broth, and the total polysaccharide content was determined using the phenol–sulfuric acid method. The soluble protein was detected by a plant total protein ELISA kit (Nuomin Kedah (Wuhan) Biotechnology Co., Ltd., Wuhan, China). Amylase activity test kits, laccase activity test kits, cellulase activity test kits, protease activity test kits, hydrogen peroxide enzyme activity test kits, superoxide dismutase activity measurement kits, and TTC dehydrogenase activity kits were provided by Shanghai Saint-bio Biotechnology Co., Ltd., Shanghai, China. Every sample had three biological replicates, and each enzyme activity index was measured in accordance with the test kit guidelines.

The unit definition of amylase activity is as follows: the catalytic production of 1 mg reducing sugar per g sample/mL liquid per minute is defined as 1 unit of enzyme activity, U.

AMY (U/g) = D × X × V1 ÷ (W × V1 ÷ V) ÷ T

AMY (U/mL) = D × X × V ÷ V1 ÷ T.

In the formula, X is the concentration (mg/mL) obtained after the absorption value △A is substituted into the standard curve, D is the dilution ratio, V is the total volume of the extraction solution (mL), V1 is the sample volume of the added reaction system (mL), W is the sample mass (g), and T is the reaction time (min).

The unit definition of laccase activity is as follows: the amount of enzyme required to oxidize 1 nmol substrate ABTS per minute per g sample/mL liquid is 1 enzyme activity unit, U.

LAC (U/g) = [△A ÷ (ε × d) × V2 × 109] ÷ (W × V1 ÷ V) ÷ T

LAC (U/mL) = [△A ÷ (ε × d) × V2 × 109] ÷ V1 ÷ T

where △A is the difference in absorbance before and after the reaction at 420 nm for 15 min; ε is the ABTS molar extinction coefficient, 36 × 10^3^ L/mol/cm; d is a 96-well plate with a light diameter of 1 cm; V, V1, V2, W, and T mean the same as in the amylase activity formula.

The cellulase activity unit definition is as follows: the catalytic production of 1 mg reducing sugar per g sample/mL liquid per minute is defined as 1 enzyme activity unit, U.

CEL (U/g) = X × V2 ÷ (W × V1 ÷ V) ÷ T

CEL (U/mL) = X × V2 ÷ V1 ÷ T

In the formula, X is the concentration (mg/mL) obtained after the absorption value △A is substituted into the standard curve, and the meanings of V, V1, V2, W, and T are the same as in the activity formula of amylase.

The protease activity unit definition: 30 °C per gram sample/per mL liquid per minute catalytic hydrolysis to produce 1 μmol tyrosine as an enzyme activity unit.

NP (U/g) = C_standard_ × ΔA_measurement_ ÷ ΔA_standard_ × V1 ÷ (W × V2 ÷ V3) ÷ T

NP (U/mL) = C_standard_ × ΔA_measurement_ ÷ ΔA_standard_ × V2 ÷ V1 ÷ T

In the formula, C_standard_ is 0.25 μmol/mL for a standard tyrosine solution; ΔA_measurement_ is the absorbance of the sample at a wavelength of 680 mm; W is the sample mass (g); V1 is the total volume of the enzymatic reaction (mL); V2 is the volume of the crude enzyme liquid (mL) added to the reaction system; V3 is the total volume of the crude enzyme liquid (mL), and T is the catalytic reaction time (min).

The catalase activity unit definition is as follows: 1 µmol H_2_O_2_ degradation per g sample per minute is 1 enzyme activity unit, U.

CAT (U/g) = [△A × V2 ÷ (ε × d) × 106] ÷ (V1 × W) ÷ T

In the formula, ε is the molar absorption coefficient of H_2_O_2_, 43.6 L/mol/cm; d is the light diameter of the colorimetric dish, 1 cm; V1, V2, W, and T mean the same as in the amylase formula.

The definition of the superoxide dismutase activity unit is as follows: when the inhibition (IR) rate of the xanthine oxidase coupling reaction system is 50%, the activity of the SOD enzyme in the reaction system is defined as an enzyme activity unit.

IR = (△A_blank_ − △A_determination_) ÷ △A blank × 100%

SOD (U/g) = [IR ÷ (1 − IR) × V2] ÷ (W × V1 ÷ V) × D

In the formula, D is the dilution ratio of the sample, and the meanings of V, V1, V2, and W are the same as those for the amylase formula.

The TTC dehydrogenase activity unit definition is as follows: Using TTC as the substrate, TTC-DH can reduce TTC and generate red 1,3,5-Triphenylformazan (TTF), which is insoluble in water. There is an absorption peak at TTF 485 nm, and the TTF content is equal to the TTC reduction amount, which represents the dehydrogenase activity.

TTC-DH[μg/(g·h)] = m/(×W × t)

where m is the TTF content (μg) of the sample extract solution obtained according to the standard curve, W is the weight of the sample (g), and t is the incubation time (h).

### 2.5. Non-Targeted Metabolomics Analysis by GC-MS and LC-MS Coupling

A metabolomic analysis was conducted on the mycelium at 10 days, 20 days, and 30 days of liquid culture, with the groups numbered sequentially as F.l-10d, F.l-20d, and F.l-30d, and each treatment included six biological replicates.

#### 2.5.1. Sample Pretreatment

Mycelia cultured for 10 d, 20 d and 30 d were selected for a metabolomic analysis, and six biological replicates were used for each treatment. The sample (30 mg) was placed into a 1.5 mL centrifuge tube, along with two small steel beads and 600 μL of 70% methanol–water solution (V:V = 7:3, with mixed internal standard, 4 µg/mL). After pre-cooling in a −40 °C freezer for 2 min, it was placed into a grinder for grinding. Ultrasonic extraction was performed in an ice-water bath for 30 min, followed by overnight incubation at −40 °C. After centrifugation for 20 min (13,000 rpm, 4 °C), 150 μL of the supernatant was aspirated and filtered using a 0.22 μm organic phase needle filter before transferring it into an LC injection vial for storage at −80 °C until the LC-MS analysis. Concurrently, 150 μL of the supernatant was transferred into a glass derivatization vial and the sample was dried using a centrifugal vacuum concentrator. Methoxyamine hydrochloride pyridine solution (80 μL, 15 mg/mL) was added to a glass derivatization vial and the oximation reaction was performed in a shaking incubator at 37 °C for 60 min. Next, 50 μL of BSTFA derivatization reagent and 20 μL of n-hexane were added, and 10 μL internal standards of fatty acids with different carbon chain lengths (C8/C9/C10/C12/C14/C16/C18/C20/C22/C24, all prepared in chloroform) were added to each and reacted at 70 °C for 60 min. After removing the sample, it was allowed to stand at room temperature for 30 min before proceeding with the GC-MS metabolomics analysis.

#### 2.5.2. LC-MS Conditions

The column used was an ACQUITY UPLC HSS T3 (100 mm × 2.1 mm, 1.8 um). The column temperature was 45 °C; the mobile phase was A-water (containing 0.1% formic acid) and b-acetonitrile; the flow rate was 0.35 mL/min; and the sample size was 3 μL. The mass range was 100–1000. The resolution was set to 70,000 for full MS scans and 17,500 for HCD MS/MS scans. The collision energies were set to 10, 20, and 40 eV. The mass spectrometer was operated as follows: spray voltage: 3800 V (+) and 3200 V (−); sheath gas flow rate: 35 arbitrary units; auxiliary gas flow rate: 8 arbitrary units; capillary temperature: 320 °C; Aux gas heater temperature: 350 °C; and S-lens RF level: 50.

#### 2.5.3. GC-MS Conditions

The GC-MS analysis was conducted using a DB-5MS capillary column (30 m × 0.25 mm × 0.25 μm, Agilent J&W Scientific, Folsom, CA, USA) with high-purity helium (≥99.999%). The flow rate was 1.0 mL/min, and the injector temperature was set at 260 °C. The injection volume was 1 μL without a shunt injection, and the solvent was delayed for 6.2 min. The initial oven temperature was 60 °C, and held at 60 °C for 0.5 min; it was then ramped up to 125 °C at a rate of 8 °C/min, to 210 °C at a rate of 5 °C/min, to 270 °C at a rate of 10 °C/min, to 305 °C at a rate of 20 °C/min, and finally held at 305 °C for 5 min. The mass spectrometer operated as follows: the electron impact (EI) ion source was maintained at 230 °C, with a quadrupole at 150 °C, and an electron energy of 70 eV. The data acquisition was performed in the full scan mode (SCAN) over a mass range of *m*/*z* 50–500.

#### 2.5.4. Data Preprocessing and Statistical Analysis

The metabolomic data analysis was performed by the Shanghai Luming Biological Technology Co., Ltd. (Shanghai, China). The raw data were processed using the metabolomics software Progenesis QI v3.0 (Nonlinear Dynamics, Newcastle, UK) for baseline filtering, peak identification, integration, retention time correction, peak alignment, and normalization. A principal component analysis (PCA) and Orthogonal Partial Least Squares Discriminant Analysis (OPLS-DA) were performed using R software package Ropl (V1.6.2). The substances were identified and screened based on the HMDB (https://hmdb.ca/), Lipidmaps (v2.3), and METLIN databases (https://metlin.scripps.edu), as well as the local LuMet-Plant3.0 database. The *p*-values were calculated using statistical tests, and the Variable Importance for the Projection (VIP) was computed using the OPLS-DA dimensionality reduction method. The statistical significance (*p*-value) was determined using a *t*-test. Criteria for differential metabolite screening were VIP > 1 and *p*-value < 0.05. The differential metabolites were used for the KEGG pathway enrichment analysis (http://www.genome.jp/kegg/, accessed on 18 June 2024). The raw data were deposited in the national genomics datacenter (https://ngdc.cncb.ac.cn/, accessed on 20 August 2024), with accession number OMIX007162.

## 3. Results

### 3.1. Changes in Four Physiological Indexes of F. luteovirens Liquid Spawn

**Figure 1:**
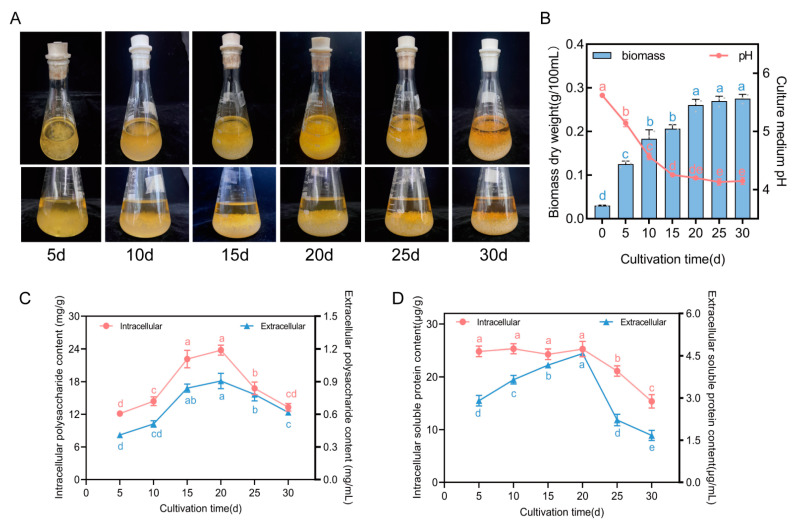
Morphology and 4 physiological indexes of *F. luteovirens* liquid spawn at different culture periods. (**A**) Morphology of *F. luteovirens* liquid spawn in shaker bottles at different periods; (**B**) mycelium biomass dry weight and pH of culture medium; (**C**) polysaccharide content; and (**D**) soluble protein content. Different lowercase letters in figure indicate significant differences at *p* < 0.05 level.

As the cultivation time increases, the mycelium in the shake flask grow continuously, and the color of the fermentation liquid visibly deepens on the 25th and 30th days (Figure 1A). The biomass increases rapidly during the first 10 days of cultivation, reaching 64% of the total mycelial biomass for the entire cycle by the 10th day. By the 20th day, the mycelial biomass reaches 0.26 ± 0.01 g/100 mL. From the 20th to the 30th day, although there is a slight increase in the mycelial biomass, no significant differences are observed between the groups (Figure 1B). The pH value of the culture medium shows a continuous decreasing trend over time, particularly during the first 15 days of cultivation, when it sharply drops from an initial value of 5.62 to reach a value of 4.25 ± 0.01; after day 15, this decrease slows down, ultimately stabilizing around approximately pH 4.1 (Figure 1B). Initially, the polysaccharide content increases with prolonged cultivation time, reaching its peak at day 20 (intracellularly 23.78 ± 0.90 mg/g, extracellularly 0.93 ± 0.11 mg/mL), followed by a continuous decline after day 20 (Figure 1C). The SP content within the cells remains relatively stable between days 5 and 20 without significant variation, ranging from 24.29 ± 4.01 to 25.28 ±1.46 μg/g; however, it rapidly decreases after day 20, while the extracellular SP content initially increases before declining again, reaching its peak at day 20 (4.58 ± 0.11 μg/mL) (Figure 1D).

### 3.2. Activity Changes in Seven Enzymes in F. luteovirens Liquid Spawn

**Figure 2:**
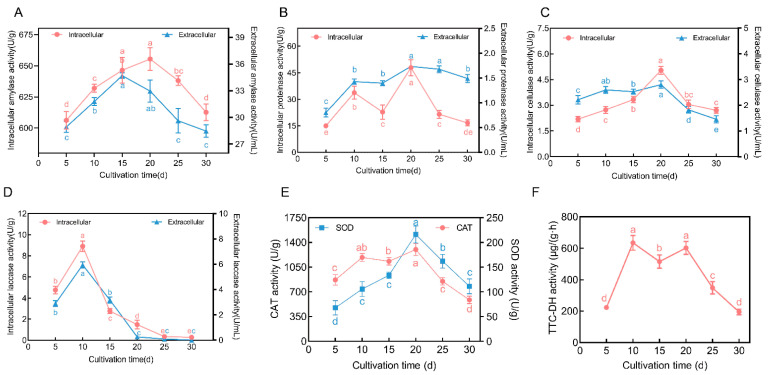
The activity of 7 enzymes of *F. luteovirens* liquid spawn during different culture periods. (**A**) Amylase activity; (**B**) protease activity; (**C**) cellulase activity; (**D**) laccase activity; (**E**) CAT and SOD activity; and (**F**) TTC-DH activity. Different lowercase letters in the figure indicate significant differences at the *p* < 0.05 level.

**Figure 3:**
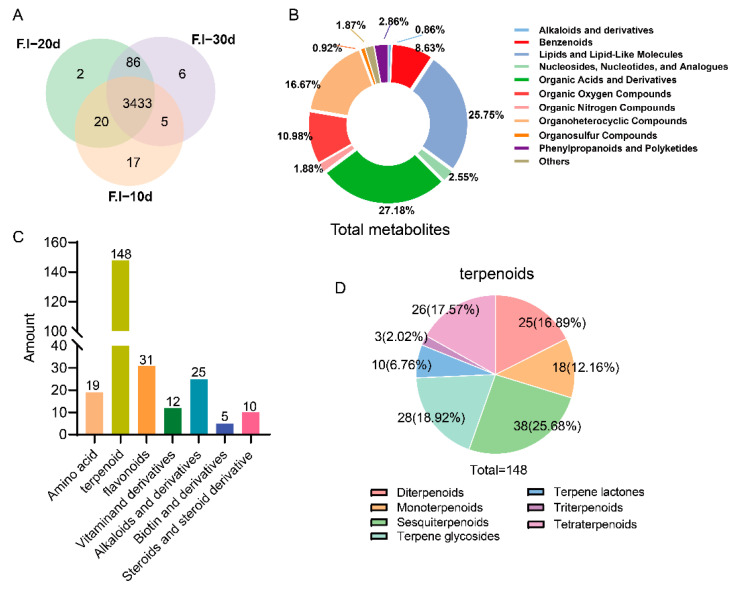
Analysis of total metabolites in mycelium of *F. luteovirens*. (**A**) Venn diagram; (**B**) classification pie chart; (**C**) statistical bar chart of number of partial bioactive compounds; and (**D**) pie chart of terpenoid classification.

Figure 2 shows that the activity of four enzymes that break down organic matter in *F. luteovirens* (amylase, protease, cellulase, and laccase) usually show a pattern of rising at the start of the cultivation process and then falling as it goes on. This trend is evident in both the intracellular and extracellular enzymatic activity, which show a roughly similar trend. Specifically, the peak activity of amylase, protease, and cellulase primarily occur on the 20th day of culture, with the only exception being the peak activity of extracellular amylase, which occurs on the 15th day. The peak activity data for these enzymes are as follows: intracellular amylase peaks at 655.45 ± 9.06 U/g and extracellular amylase at 34.69 ± 0.32 U/mL; intracellular protease peaks at 47.87 ± 4.50 U/g and extracellular protease at 1.73 ± 0.25 U/mL; and intracellular cellulase peaks at 5.04 ± 0.23 U/g and extracellular cellulase at 2.81 ± 0.14 U/mL. In contrast, the maximum activity of laccase occurs earlier (Figure 2D), reaching an intracellular activity of 8.91 ± 0.49 U/g and an extracellular activity of 5.96 ± 0.25 U/mL on the 10th day of culture. However, it is noteworthy that after 20 days of culture, the activity of laccase almost diminishes to undetectable levels. The activity of the three oxidoreductases also generally exhibit an initial increase followed by a decrease. The activity of the CAT and SOD enzymes peak on the 20th day, with the former being 1299.49 ± 85.18 U/g (Figure 2E) and the latter 216.07 ± 17.17 U/g (Figure 3B), which are significantly higher than at other times. TTC-DH activity reaches its maximum value on the 10th day (635.12 ± 46.98 μg/(g·h). It decreases by the 15th day, but significantly increases again on the 20th day (602.25 ± 40.87 μg/(g·h). After the 20th day, TTC-DH activity rapidly decreases (Figure 2F).

### 3.3. Comprehensive Analysis of Primary Metabolites of F. luteovirens Liquid Spawn

**Figure 4:**
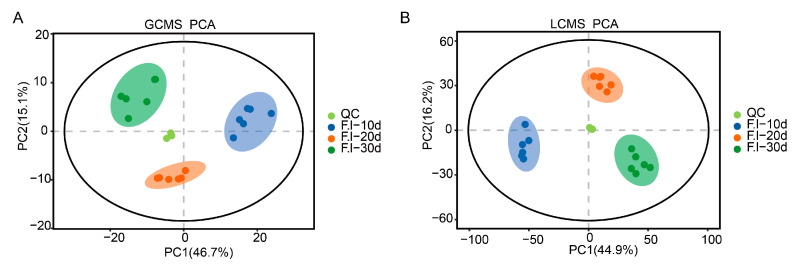
PCA score plot. (**A**) Graph of PCA score for GCMS assay; (**B**) graph of PCA score for LCMS assay. Points of the same color represent six replicas.

As shown in Figure 4, a total of 3569 metabolites were detected in the *F. luteovirens* mycelium (Figure 3A), including 970 organic acids and their derivatives; 919 lipids and lipid-like molecules; 595 heterocyclic compounds; 392 organic oxygen compounds; 308 benzene compounds; 102 phenylpropanoids and polyketides; 91 nucleosides, nucleotides, and analogues; 67 organic nitrogen compounds; 33 organic sulfur compounds; 25 alkaloids and their derivatives; and 67 other compounds (Table S1, Figure 3B). The mycelium of *F. luteovirens* shared 3433 metabolites across the three periods, with the unique metabolites numbering 17, 2, and 6 at 10 d, 20 d, and 30 d, respectively (Figure 3A), indicating that the overall variety of metabolites did not change significantly during the growth process. Among these, the metabolites shared between 20 d and 30 d were the most numerous, totaling 3519, suggesting that the metabolic levels of mycelial growth at 20 d and 30 d were more similar (Figure 3B). During the classification process of all the metabolites, a total of 19 common amino acids constituting proteins were identified, along with 10 vitamins, five biotins, 26 alkaloid compounds, 31 flavonoid compounds, 10 steroid compounds, and 148 terpenoid compounds (Table S2, Figure 3C). Among these terpenoids, Sesquiterpenoids were the most abundant, accounting for 25.68%, followed by terpene glycosides (18.92%), triterpenoids (17.58%), and diterpenoids (16.89%) (Figure 3D). These data indicate that the mycelium of *F. luteovirens* contains a rich array of metabolites and diverse bioactive components, particularly terpenoids.

### 3.4. Principal Component Analysis (PCA) of Mycelium at Different Culture Stages

To further investigate the differences in metabolite accumulation in the *F. luteovirens* mycelium, a PCA was conducted on the metabolites of *F. luteovirens* at three different stages. The results from the GC-MS data show that the first principal component accounted for 46.7% of the total variability in the dataset, whereas the second principal component explained 15.1% (Figure 4A). For the LC-MS data, the first principal component represented 44.9% of the total variability, and the second principal component accounted for 16.2% (Figure 4B). The PCA results indicate that the samples clustered within their respective groups and were clearly separated between groups, suggesting significant differences in the metabolites in the mycelium at different culture stage.

### 3.5. Orthogonal Partial Least Squares (OPLS-DA) Analysis

**Figure 5:**
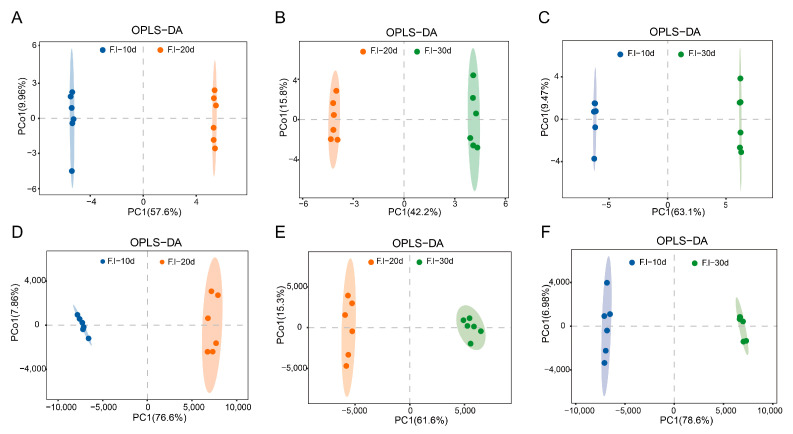
OPLS-DA score plot. (**A**) Plot of OPLS-DA scores for F.l-10d vs. F.l-20d using GCMS analysis; (**B**) plot of OPLS-DA scores for F.l-20d vs. F.l-30d using GCMS analysis; (**C**) plot of OPLS-DA scores for F.l-10d vs. F.l-30d using GCMS analysis; (**D**) plot of OPLS-DA scores for F.l-10d vs. F.l-20d using LCMS analysis; (**E**) plot of OPLS-DA scores for F.l-20d vs. F.l-30d using LCMS analysis; (**F**) plot of OPLS-DA scores for F.l-10d vs. F.l-30d using LCMS analysis. Points of same color represent six replicas.

**Figure 6:**
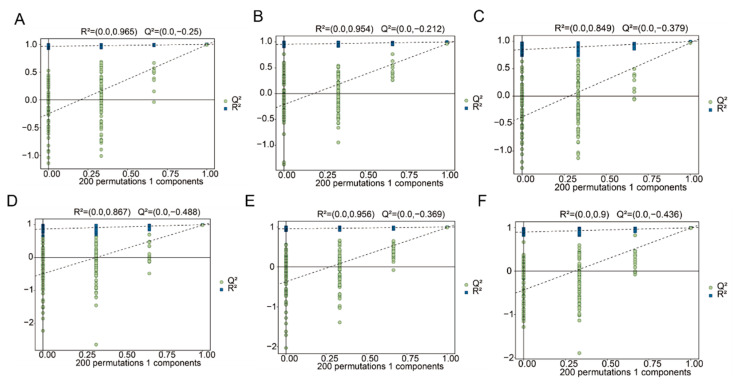
OPLS permutation test plot. (**A**) Plot of permutation test for F.l-10d vs. F.l-20d using GCMS analysis; (**B**) plot of permutation test for F.l-20d vs. F.l-30d using GCMS analysis; (**C**) plot of permutation test for F.l-10d vs. F.l-30d using GCMS analysis; (**D**) plot of permutation test for F.l-10d vs. F.l-20d using LCMS analysis; (**E**) plot of permutation test for F.l-20d vs. F.l-30d using LCMS analysis; (**F**) plot of permutation test for F.l-10d vs. F.l-30d using LCMS analysis.

The OPLS-DA model is a supervised discriminant analysis statistical method that can effectively eliminate irrelevant influences and screen differential metabolites [25]. In the OPLS-DA model, R2X (cum) and R2Y (cum) represent the explanatory rates of the constructed model for the X and Y matrices, respectively, and Q2 indicates the predictive ability of the model. The OPLS-DA method was further used to analyze the differential metabolic products of the *F. luteovirens* mycelia cultured for 10, 20, and 30 days. For each comparison group, R2X, R2Y, and Q2 were all greater than 0.9 and close to 1 (Figure 5), indicating that the OPLS-DA model had a good predictive performance. The model, in this study, expressed the differences and distinctions in the metabolic products of the *F. luteovirens* mycelia at different culture periods. A permutation test was used to validate the model to avoid overfitting the supervised model during the modeling process. After 200 cross-validations, as the R2 and Q2 of the random models gradually decreased (Figure 6), it was demonstrated that the established model had good reliability without overfitting, and the separation of the metabolites between the groups was statistically significant.

### 3.6. Statistical Analysis of Differential Metabolites

Upon identification, 492 differential metabolites from the mycelia of *F. luteovirens* across the three culture durations were screened. Between F.l-10d and F.l-20d, there were 299 differential metabolites, with 88 upregulated and 211 downregulated; between F.l-20d and F.l-30d, there were 291 differential metabolites, with 120 upregulated and 171 downregulated; and between F.l-10d and F.l-30d, there were 381 differential metabolites, with 122 upregulated and 259 downregulated (Table 1). The categories with significant differences included carboxylic acids and their derivatives (69 species), fatty acyl groups (43 species), organic oxygen compounds (42 species), and lipid compounds (32 species). Carboxylic acids and their derivatives were the most numerous class of differential metabolites, with amino acids and their metabolites playing the primary role. Among the secondary metabolites, flavonoids, phenolics, and alkaloids showed relatively fewer differential metabolites at the various stages of mycelial culture. However, it is noteworthy that isoprenoid lipids (mainly terpenes) had a higher number of differential metabolites (≥9) across all the periods, indicating that the types and contents of terpenoid substances changed significantly with the culture time.

### 3.7. Comparative Analysis of Differential Metabolites

**Figure 7:**
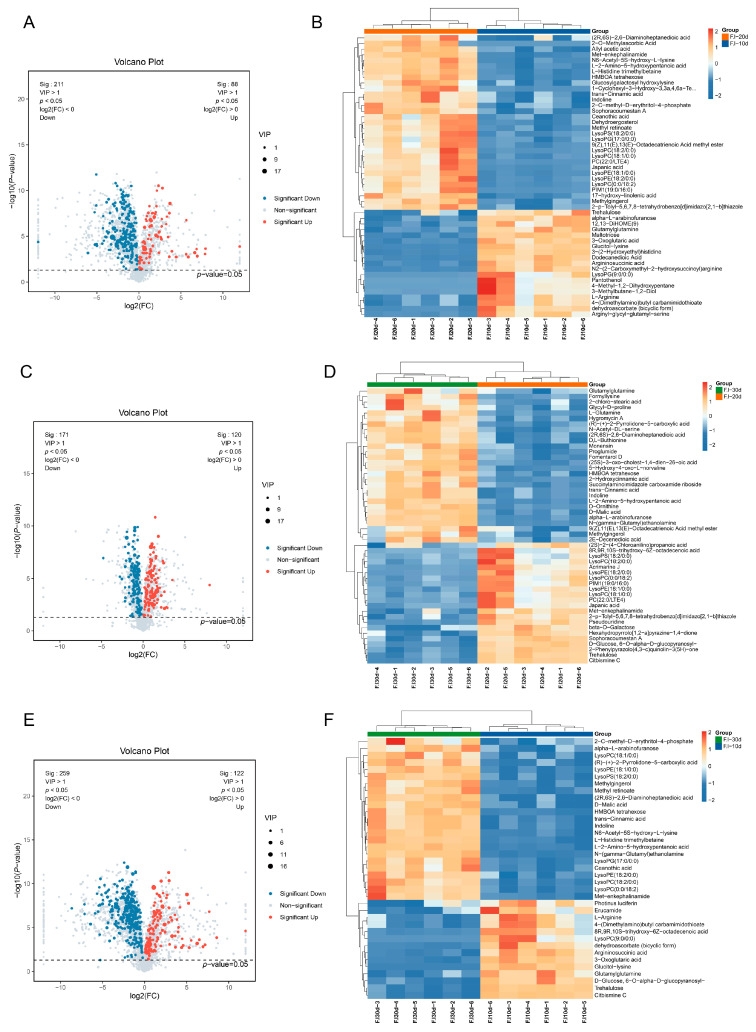
Differential metabolite volcano map and differential metabolite cluster heat map. (**A**) Fl-10d vs. Fl-20d differential metabolite volcano map; (**B**) Fl-10d vs. Fl-20d differential metabolite cluster heat map; (**C**) Fl-20d vs. Fl-30d differential metabolite volcano map; (**D**) FL-20d vs. FL-30d differential metabolite cluster heat map; (**E**) Fl-10d vs. Fl-30d differential metabolite volcano map; and (**F**) Fl-10d vs. Fl-30d differential metabolite cluster heat map.

Throughout the culture period, the mycelium gradually transitioned from a rapid growth phase to a slow growth phase, with continuous changes in the types and quantities of metabolites. Using a *p*-value of less than 0.05 and a VIP score greater than 1, the significantly differentially expressed metabolites (DEMs) were filtered out during pairwise comparisons. These DEMs are represented in volcano plots (Figure 7A,C,E). To more intuitively display the differences in metabolite expression in the mycelium of *F. luteovirens* at different culture times, a hierarchical clustering of the expression of the significantly different metabolites in each comparison group was performed according to VIP Top50 (Figure 7B,D,F).

In the comparison of groups 10d and 20d, the VIPtop50 DEMs were mainly categorized into amino acids, metabolic intermediates, sugars, phospholipids, etc. There were 18 chemical components that had a greater relative content in the 10d sample compared to the 20d sample. The three most significantly differentially expressed metabolites were Maltotriose, Glucitol-lysine, and 3-(2-Hydroxyethyl) histidine (Table S4). Other upregulated metabolites included Trehalulose, L-Arginine, dehydroascorbate (bicyclic form), Pantothenol, etc. (Figure 7B). In addition, among all the DEMs, we also noticed that some secondary metabolites were significantly upregulated, such as the alkaloid compound Mulberrofuran E and the terpene compound Sterebin B (Table S3). There were 32 chemical components with a lower relative content in the 10d sample than in the 20d sample, including eight phospholipid molecules, among which LysoPE (18:2/0:0) and LysoPC (0:0/18:2) were the most significant; there were seven amino acids and peptides analogues, among which Met-enkephalinamide and L-Histidine trimethylbetaine were the most significant (Table S4). Other downregulated metabolites included various organic acids, such as trans-Cinnamic acid, Ceanothic acid, Japanic acid, etc. (Figure 7B).

In the comparison of groups 20d and 30d, the VIPtop50 DEMs were mainly classified into phospholipids, sugars and sugar alcohols, amino acids and peptides, organic acids, and so on. There were 22 chemical components in which the relative content was higher in the 20d sample compared to the 30d sample. These components included eight glycerophospholipid molecules, with the biggest fold change being that for PIM1 (19:0/16:0), PC (22:0/LTE4), LysoPE (18:2/0:0), among others (Table S4). For the rest of the upregulated DMEs, the primary metabolites included Trehalulose, D-Glucose, 6-O-alpha-D-glucopyranosyl-, Pseudouridine, etc. The secondary metabolites included Sophoracoumestan A, Citbismine C, Japanic acid, and Acrimarine J, etc. (Figure 7D). There were 28 chemical components with a lower relative abundance in the 20d than in the 30d, among which there were 14 amino acids, peptides, and analogues, with the highest fold change being that for Proglumide and D-Ornithine (Table S4). Other metabolites that exhibited downregulation include alpha-L-arabinofuranose, glutamylglutamine, and formyllysine, as well as certain secondary metabolites, such as D-malic acid, trans-Cinnamic acid, and Fomentarol D (Figure 7D). Furthermore, we observed a notable decrease in the expression of several sugars, including D-Galactose, Glucose, Erythritol, D-Gulose, and D-Psicose, as well as in several amino acids, such as Pyroglutamic Acid, Ornithine, L-Alanine, and L-Proline (Table S3).

In the comparison of groups 10d and 30d, the DEMs in the VIPtop50 were mainly classified into amino acids and their derivatives, phospholipids, sugars and sugar alcohols, etc. There were 16 chemical components with a higher relative content in the 10d sample than in the 30d sample, among which the three most significantly differentially expressed metabolites were Citbismine C, Trehalulose, and Glucitol-lysine (Table S4), and other upregulated metabolites also included L-Arginine, Trehalulose, Glucitol-lysine, Argininosuccinic acid, etc. (Figure 7F). The chemical components with a lower relative content in the 10d sample than in the 30d sample included 34 kinds, mainly amino acid derivatives and fatty acid derivatives, among which the three most significantly differentially expressed metabolites were N-(gamma-Glutamyl)ethanolamine, L-2-Amino-5-hydroxypentanoic acid, and alpha-L-arabinofuranose (Table S4); other downregulated metabolites also included D-Lysine, Glycyl-D-proline, and Indoline; and significantly downregulated secondary metabolites included trans-Cinnamic acid, Methylgingerol and D-Malic acid (Figure 7F).

**Figure 8:**
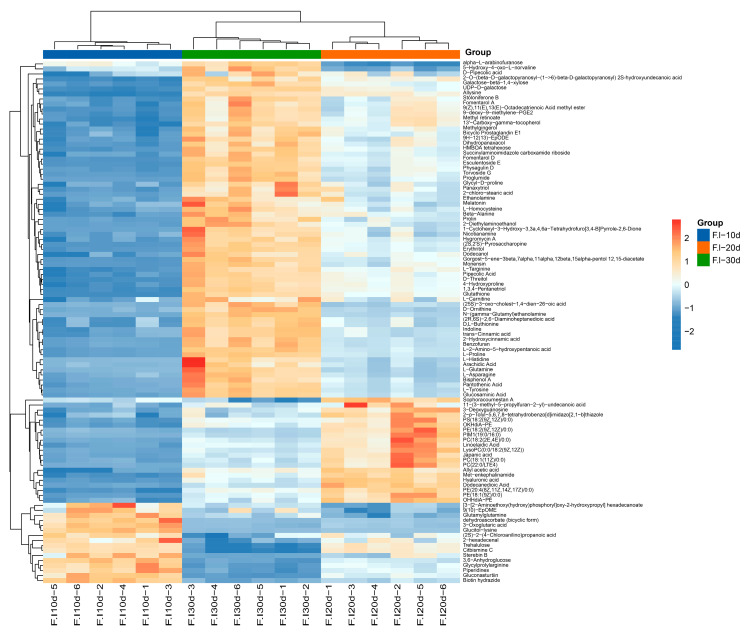
Clustering heat map of differential metabolites common to all three sample groups.

From the 104 common DEMs found across all periods, 65 showed a continuous increase in expression levels with increasing culture time. Among these, amino acids, peptides, and similar compounds accounted for 40%; fatty acids and conjugates, carbohydrates and carbohydrate conjugates, ergostane compounds, and fatty alcohols accounted for 8%, 6%, 5%, and 5%, respectively (Table S5). The metabolites with higher contents included L-Glutamine, L-Histidine, L-Tyrosine, L-Proline, Nicotianamine, Arachidic Nicotianamine, Arachidic Acid, D-Threitol, and HMBOA tetrahexose, as well as secondary metabolic products, such as Fomentarol A, Dihydropanaxaco, and Panaxasol D (Figure 8). Only 13 metabolites showed a continuous decrease in content with increasing culture time, including trehalose, dehydroascorbate (bicyclic form), and 3-Oxoglutaric acid. A total of 21 metabolites first increased and then decreased in content, including 11 glycerophospholipid compounds, such as Phosphatidylcholine LysoPC (18:1/0:0); five fatty acyl compounds, such as Dodecanedioic Acid; and five other compounds (Figure 8).

### 3.8. KEGG Pathway Enrichment Analysis of Differential Metabolites

**Figure 9:**
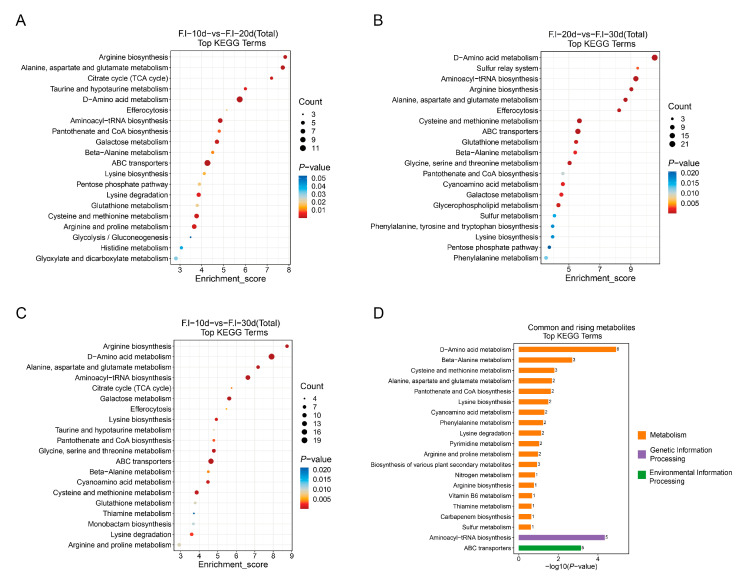
KEGG enrichment analysis map of differential metabolites among groups. (**A**) Fl-10d vs. Fl-20d KEGG enrichment analysis; (**B**) Fl-20d vs. Fl-30d KEGG enrichment analysis; (**C**) Fl-10d vs. Fl-30d KEGG enrichment analysis; (**D**) KEGG enriched metabolic pathway map of differential metabolites common to three groups and with increasing content over culture time.

The differential metabolites were matched with the KEGG database and subjected to a metabolic enrichment analysis (Figure 9, Table S3). Inter-group comparisons at various time points revealed that the significantly enriched metabolic pathways were primarily amino acid metabolism pathways, especially D-amino acid metabolism.

In the comparison of groups F.l-10d and F.l-20d (Figure 9A), 64 pathways were enriched, among which 19 metabolic pathways were significantly different (*p* < 0.05). The top-five metabolic pathways with the highest degrees of enrichment were as follows: 1. arginine biosynthesis; 2. alanine, aspartate, and glutamate metabolism; 3. citrate cycle (TCA cycle); 4. taurine and hypotaurine metabolism; and 5. D-amino acid metabolism. Among the top-ten metabolic pathways with the most differential metabolite enrichment, seven were amino acid metabolism pathways, namely D-amino acid metabolism; cysteine and methionine metabolism; arginine and proline metabolism; alanine, aspartate, and glutamate metabolism; galactose metabolism; lysine degradation; and arginine biosynthesis. The remaining three were ABC transporters, aminoacyl-tRNA biosynthesis, and amino sugar and nucleotide sugar metabolism.

In the comparison of groups F.l-20d and F.l-30d (Figure 9B), 61 pathways were enriched, among which 24 metabolic pathways were significantly different (*p* < 0.05). The top-five metabolic pathways with the highest degrees of enrichment were as follows: 1. D-amino acid metabolism, 2. sulfur relay system, 3. aminoacyl-tRNA biosynthesis, 4. arginine biosynthesis, and 5. alanine, aspartate, and glutamate metabolism. Among the top-ten metabolic pathways with the most differential metabolite enrichment, five were amino acid metabolism pathways, namely D-amino Acid metabolism; cysteine and Methionine metabolism; alanine, aspartate, and glutamate metabolism; arginine biosynthesis; and glycine, serine, and threonine metabolism. The remaining five were aminoacyl-tRNA biosynthesis, ABC transporters, efferocytosis, glutathione metabolism, and glycerophospholipid metabolism.

In the comparison of groups F.l-10d and F.l-30d (Figure 9C), 67 pathways were enriched, among which 26 metabolic pathways were significantly different (*p* < 0.05). The top-five metabolic pathways with the highest degrees of enrichment were as follows: 1. arginine biosynthesis; 2. D-amino acid metabolism; 3. alanine, aspartate, and glutamate metabolism; 4. aminoacyl-tRNA biosynthesis; and 5. citrate cycle (TCA cycle). Among the top-ten metabolic pathways with the most differential metabolite enrichment, seven were amino acid metabolism pathways, namely D-amino acid metabolism; arginine biosynthesis; alanine, aspartate, and glutamate metabolism; glycine, serine, and threonine metabolism; cysteine and methionine metabolism; cyanoamino acid metabolism; and lysine biosynthesis. The remaining three were aminoacyl-tRNA biosynthesis, ABC transporters, and galactose metabolism.

In the KEGG enrichment analysis of the differential metabolites that were common across all three periods (Figure 9D) and showed a continuous increase in content, a total of 36 pathways were enriched; of these, eight metabolic pathways were significantly different (*p* < 0.05), namely D-amino acid metabolism; aminoacyl-tRNA biosynthesis; ABC transporters; beta-alanine metabolism; cysteine and methionine metabolism; alanine, aspartate, and glutamate metabolism; pantothenate and coenzyme A biosynthesis; and lysine biosynthesis. Other metabolic pathways with a high degree of enrichment included vitamin B6 metabolism, the biosynthesis of various plant secondary metabolites, and so on.

## 4. Discussion and Conclusions

### 4.1. Analysis of Changes in Enzyme Activity of F. luteovirens Liquid Spawn

Under liquid culture conditions, the growth and metabolism of *F. luteovirens* mycelium are closely related to the culture duration, with enzyme activity exhibiting dynamic changes throughout the cultivation process. It was observed that the activity of seven enzymes all followed a trend of initially increasing and then decreasing (Figure 2). Specifically, the activity of amylase, protease, cellulase, SOD, and CAT peaked around the 20th day, while the activity peaks for laccase and TTC-DH occurred on the 10th day. However, one clear aspect is that after 20 days of cultivation, the activity of both hydrolytic and oxidoreductase enzymes began to diminish significantly. In conjunction with the changes in mycelial biomass and the pH of the culture medium (Figure 1B), it can be inferred that during the first 20 days of cultivation, the mycelium is in a rapid growth phase, demanding higher levels of nutrients. The increased hydrolytic enzyme activity facilitates the absorption and utilization of the macromolecules in the culture medium. In the later stages, as the mycelium enters a stable growth phase with a slowdown in the cell proliferation rate and a reduced demand for nutrients, the activity of the hydrolytic enzymes decreases correspondingly. Concurrently, as the nutrients in the culture medium are gradually consumed, the activity of the hydrolytic enzymes decreases when these substrates are depleted. Studies on the liquid cultures of *Phellinus igniarius* and *Naematelia aurantialba* have found that enzyme activity related to the decomposition of starch and lignocellulose first increase and then decrease with the cultivation time [26,27]. SOD and CAT are pivotal components of the antioxidant defense system of organisms, playing a critical role in preventing oxidative stress to cellular membrane lipids and delaying cellular senescence [28]. In this study, the activity levels of CAT and SOD peaked on the 20th day of culture (Figure 2E), coinciding with the onset of a stationary phase in the mycelial biomass. This indicates that reactive oxygen species (ROS) generated within the cells can be effectively neutralized at this time. However, as the duration of the culture extends, the cellular capacity to metabolize ROS gradually diminishes, leading to a decline in the vitality of *F. luteovirens* liquid spawn. Previous research has established a correlation between TTC-DH activity and various enzymatic activities, suggesting that higher TTC-DH activity corresponds to increased cell vitality and, in the case of edible mushrooms, higher yields [12,13]. In this experiment, TTC-DH activity was notably high between 10 and 20 days, which, to some extent, indicates the stronger vitality of *F. luteovirens* spawned in liquid culture during this period.

### 4.2. Analysis of Metabolic Products of F. luteovirens Mycelium

In this study, a non-targeted metabolomics approach utilizing both GC-MS and LC-MS technologies was employed to identify a total of 3569 metabolites from the *F. luteovirens* mycelium. This included 723 amino acids and their derivatives, encompassing all 19 proteinogenic amino acids except leucine (although various leucine derivatives were detected), as well as 919 lipid compounds (Table S1). These findings suggest that *F. luteovirens* mycelia have the potential to serve as an alternative source of dietary proteins, lipids, fatty acids, and other components traditionally derived from fruiting bodies. Common secondary metabolites, such as flavonoids, terpenoids, and alkaloids, found in edible fungi have also been detected in large quantities in the mycelium of *F. luteovirens*, with the number of terpenoid compounds reaching up to 148 (Table S2). Terpenoid compounds exhibit a wide range of biological activities, particularly showing significant anticancer activity and anti-inflammatory effects [17]. In addition, a secondary metabolite, named ergothioneine (ET), has been discovered. ET is a natural antioxidant [29] and has been widely applied across various industries, including food, functional cosmetics, and pharmaceuticals [30]. These research findings indicate that the mycelium of *F. luteovirens* is a valuable reservoir of nutrients and bioactive components, with immense potential for development and application prospects.

This work examined the DMEs of *F. luteovirens* mycelia at three different time points: 10, 20, and 30 days of culture. A total of 492 distinct metabolites were discovered and recorded in Table S2. The results demonstrate that the kinds and amounts of amino acids, nucleotides, fatty acyl groups, and lipid molecules typically changed, reflecting the continual synthesis and degradation of the basic substances essential for mycelial growth. Amino acids are the primary building elements of proteins. They serve as an organic nitrogen source, providing important nutrients, and also operate as compounds that regulate the life processes of organisms. Numerous studies have shown that the addition of exogenous amino acids considerably enhances the growth of edible fungi. The study conducted by Hang et al. [31] discovered that the introduction of external arginine can greatly enhance the development of the mycelium of *Hypsizygus marmoreus*. This confirmed that external amino acids can act as the primary sources of nourishment. In a similar vein, Chang et al. [32] conducted experiments that revealed that the addition of exogenous alanine, serine, and asparagine to a culture medium enhanced the growth of *Lentinus edods* mycelium, making it more efficient. Furthermore, the mycelium cultured with an amino acid mixture exhibited a significant increase in pyruvic acid and protein content. In our study, we focused special attention on four specific amino acids: L-Glutamine, L-Histidine, L-Tyrosine, and L-Proline. The levels of these amino acids consistently rose throughout the cultivation process (Figure 8). Thus, we propose that these amino acids can serve as exogenous supplements in the medium, offering a valuable resource for the cultivation of *F. luteovirens* and the improvement of the issue of sluggish mycelium growth. In addition, carbohydrates not only provide energy for the mycelia, but also help maintain the stability of the cell structure and participate in regulating the growth and development of fungi [33]. In this study, the trehalose content was highest at the initial stage of culture (10 days) and gradually decreased with the extension of the culture time (Figure 7B), suggesting that trehalose may play an important role in promoting the rapid growth phase of *F. luteovirens* mycelia. At the end of the culture (30 days), the contents of D-Galactose, Glucose, Erythritol, D-Gulose, and D-Psicose increased compared to those at 20 days, while the content of polysaccharides in the fermentation broth was reduced, indicating that polysaccharides might be re-decomposed into monosaccharides for cell utilization.

Throughout the entire culture cycle, mycelia simultaneously produced and accumulated a wealth of secondary metabolites. For example, after 10 days of culture, the contents of Mulberrofuran E and Sterebin B were higher (Figure 7B,F). Mulberrofuran E showed stronger in vitro α-glucosidase inhibitory activity [34]. Sterebin B, a diterpene compound, is thought to exhibit the typical effects of terpenoids, such as antioxidant, antibacterial, and antiviral activities [35]. At 20 days of culture, we detected relatively high levels of two substances, Sophoracoumestan A and Citbismine C (Figure 7B,D). Between these, Sophoracoumestan A is classified as an isoflavone compound, which has significant antioxidant and antitumor biological activities, and has the potential to be developed as a chemotherapy drug for aggressive cancers [36]. Citbismine C is a diacridone alkaloid. Studies have tested the cytotoxicity of five diacridone alkaloids, including Citbismine C, to human leukemia HL-60 cells, and found that these compounds have the potential to act as anticancer agents [37]. After 30 days of culture, the contents of trans-cinnamic acid and malic acid significantly increased (Figure 7D,F). Both compounds are effective free radical scavengers, and in the medical field, trans-cinnamic acid can reduce the production of inflammatory mediators and be used in combination with other medications to enhance antimicrobial efficacy [38]; malic acid has potential applications in the treatment of liver disease, diabetes, immunodeficiency, and can also mitigate the toxic effects of anticancer drugs on normal cells [39]. In summary, *F. luteovirens* mycelia has the potential to be developed for the efficient production of its functional active ingredients.

### 4.3. Differential Metabolic Pathway Analysis

From the comparative analysis of the different groups, our findings reveal that there are at least seven metabolic pathways related to amino acid metabolism in the top-20 pathways listed by the Enrichment Score (Figure 9A–C). It is worth mentioning that the pathways related to Cysteine and Methionine metabolism; Lysine biosynthesis; Alanine, Aspartate, and Glutamate metabolism; and Arginine biosynthesis consistently showed high enrichment in all the groups. This observation highlights the crucial significance of these particular amino acid metabolic pathways in the growth and development of mycelia. Extensive research has been carried out on this topic. For instance, studies on the plant-harming fungus *Alternaria alternata* have shown that mutants of three genes—MetB, MetC, and MetX—that are closely linked to methionine metabolism do not grow as well as they should. These mutants display not only decreased growth rates on culture media, but also notable changes in their morphology, resistance to reactive oxygen species, and pathogenicity [40]. Lysine, an essential amino acid, is biosynthesized through the α-aminoadipate pathway in higher fungi. A study on *Arthrobotrys oligospora* revealed that the homologous recombination-mediated knockout of the Aoaar gene, which codes for α-aminoadipate reductase, has a big impact on the fungus’s ability to grow, make spores, and avoid being eaten by nematodes. This emphasized the critical function of the Aoaar gene in lysine biosynthesis and overall fungal physiology [41]. Another study on *Metarhizium anisopliae* suggested that amino acid metabolism, particularly involving glutamate, aspartate, serine, glycine, arginine, and leucine, significantly influences a fungus’s conidiation mechanism. Comparing normal and degenerate strains revealed that metabolic differences in these amino acids are likely key factors contributing to variations in conidiation capacity [42]. Furthermore, our study finds D-amino acid metabolic pathways to be the most abundant category of DEMs across all the compared sets (Figure 9). It is widely acknowledged that bacteria create a variety of D-amino acids required for the synthesis of bacterial cell wall peptidoglycan [43]. At present, advances in amino acid chiral analysis have revealed the presence of D-amino acids not just in bacteria, but also in humans, other animals, plants, and microbes, where they serve a variety of biological purposes [44]. Nonetheless, research into D-amino acids in fungi remains limited. Given our existing understanding, it is only possible to speculate that D-amino acids play a regulatory role in *F. luteovirens* growth and development. Nevertheless, it should be noted that their isomeric compounds often display similar retention behavior during mass spectrometry analysis, making them difficult to distinguish accurately. Consequently, utilizing metabolomic data to analyze these isomers presents certain challenges. If we want a deeper investigation into the D-amino acids in *F. luteovirens*, more precise detection methods will be required to characterize and quantify these metabolites accurately, thereby elucidating their roles and mechanisms in biological processes.

In the comparative groups 10d vs. 20d and 10d vs. 30d (Figure 9A,C), multiple metabolic pathways related to amino acids were significantly downregulated. These pathways included arginine biosynthesis; the metabolism of alanine, aspartate, and glutamate; and lysine biosynthesis and degradation. These changes may be associated with the limited availability of nutrients and the slowdown in cell division as the mycelium enters a stable growth phase. In the later stages of the culture, due to the extensive consumption of external nutrients, the *F. luteovirens* mycelia began to utilize certain non-essential amino acids, such as alanine, aspartate, and glutamate, as sources of carbon and nitrogen. Concurrently, the synthesis of arginine increased; arginine is an important nitrogen storage nutrient [45], and plays a crucial role in maintaining growth and responding to acidic environments [46]. Simultaneously, the balance between lysine synthesis and degradation became particularly important. When L-lysine accumulates to a certain level within a cell, it can inhibit the activity of key enzymes, thereby affecting cell growth [47,48]. These metabolic adjustments help the mycelium to adapt to changes in nutrient availability and maintain normal growth metabolism. In the comparison of groups 20d vs. 30d (Figure 9B), Galactose metabolism, fructose and mannose metabolism, fatty acid degradation, and fatty acid biosynthesis were significantly upregulated. These metabolic pathways provide energy and carbon sources for cells and indirectly affect secondary metabolite synthesis in the mycelium. Metabolites with consistently elevated relative levels throughout the culture period were eventually enriched in pathways, such as pantothenic acid and coenzyme A biosynthesis, vitamin B6 metabolism, and the biosynthesis of plant secondary metabolites. Pantothenic acid is a precursor of vitamin B5, suggesting that, with increasing culture time, the metabolism of certain vitamins and secondary metabolites is continuously strengthened.

## 5. Summary

The artificial cultivation of *F. luteovirens* has consistently posed difficulties, due to the sluggish growth of its mycelium under laboratory conditions. This has hampered the progress and usage of this valuable edible mushroom resource. Through the utilization of a liquid culture, we successfully harvested the mycelium in this experiment, resulting in a higher quantity of biomass within a reduced timeframe. During the experiment, it was seen that after approximately 20 days of cultivation, the biomass of the *F. luteovirens* mycelium reached a stable state, and after this time, the activity of numerous enzymes markedly declined. Thus, we assert that a 20-day period is the most advantageous duration for harvesting the liquid spawn. The metabolomics findings indicate that the mycelium of *F. luteovirens* contains a high concentration of active compounds, particularly terpenoids. The further analysis of the mycelial metabolite changes during liquid culture revealed that the types and contents of metabolites showed diverse trends at different stages of culture. Carboxylic acids and their derivatives were the most abundant among the metabolites that showed differential expression. Amino acids and their metabolites played a vital part in this process. Terpenoid compounds exhibited the greatest number of differential metabolites among the secondary metabolites during the different culture phases. Furthermore, as the culture advanced, the accumulation of diverse organic acids continued. During the growth phase of the *F. luteovirens* mycelium, the most significant alterations occurred in amino acid metabolism, with various amino acid metabolic pathways interacting to support cell growth. These findings enhance our understanding of the metabolic composition and changes in the *F. luteovirens* mycelium, providing theoretical support for understanding the metabolic pathways of mycelial growth, and offering a theoretical foundation for liquid culture and the potential functional component development and utilization of *F. luteovirens.*

## Figures and Tables

**Table 1:** Statistical table of differential metabolite species.

Types of Metabolites	F.l-10d vs. F.l-20d			F.l-20d vs. F.l-30d			F.l-10d vs. F.l-30d		
	Total	UP	Down	Total	UP	Down	Total	UP	Down
Azacyclic compounds	1	0	1	5	2	3	6	2	4
Furans	5	1	4	7	4	3	7	4	3
Nucleotides and derivatives	8	1	7	11	6	5	11	3	8
Flavonoids	3	2	1	1	1	0	2	2	0
Harmala alkaloids	3	1	2	3	3	0	3	2	1
Pteridines and derivatives	5	2	3	1	0	1	5	2	3
Glycerophospholipids	32	10	22	27	19	8	29	8	21
Lactones	4	2	2	1	0	1	4	2	2
Organic phosphoric acids and derivatives	3	1	2	3	2	1	3	1	2
Phenols	3	1	2	1	0	1	5	2	3
Benzene and substituted derivatives	11	4	7	9	4	5	13	6	7
Biotin and derivatives	1	1	0	1	1	0	1	1	0
Carboxylic acids and derivatives	69	22	47	85	21	64	95	28	67
Cinnamic acids and derivatives	3	0	3	3	1	2	2	0	2
Benzodiazepines	4	1	3	2	1	1	5	2	3
Fatty acyls	43	6	37	43	21	22	52	12	40
Hydroxy acids and derivatives	4	0	4	4	2	2	7	1	6
Indoles and their derivatives	3	0	3	4	1	3	3	0	3
Keto acids and derivatives	3	1	2	2	1	1	4	2	2
Organonitrogen compounds	6	1	5	7	2	5	7	2	5
Organooxygen compounds	42	18	24	38	17	21	53	22	31
Prenol lipids	12	1	11	9	4	5	17	3	14
Steroids and steroid derivatives	14	2	12	13	1	12	25	2	23
Other	17	10	17	11	6	5	22	13	9
Total	299	88	221	291	120	171	381	122	259

## Data Availability

The original contributions presented in the study are included in the article/Supplementary Material, further inquiries can be directed to the corresponding author.

## Supplementary Materials

The following supporting information can be downloaded at: https://www.mdpi.com/article/10.3390/jof10090618/s1. Table S1. all metabolite data; Table S2. bioactive compounds; Table S3. Differential expression of metabolite data; Table S4. DMEs-VIPtop50; Table S5. Common metabolite heat map.
